# Engaging traders in strengthening seed systems in Tanzania: key drivers for selling grains of improved varieties of sorghum, common beans and groundnuts

**DOI:** 10.1007/s44279-025-00413-2

**Published:** 2025-11-10

**Authors:** Dennis Ong’or, Justus Ochieng, Atupokile Mwakatwila, Mercy Mutua, Radegunda Kessy, Sylvia Kelemera, Paul Aseete, Emmanuel Mwenda, Joachim Madeni, Edith Kadege, Jean Claude Rubyogo

**Affiliations:** 1 International Center for Tropical Agriculture (CIAT), P.O. Box 823, Nairobi, Kenya; 2 International Center for Tropical Agriculture (CIAT), P.O. Box 2704, Arusha, Tanzania; 3 Makerere University, P.O. Box 7062, Kampala, Uganda; 4 Tanzania Agricultural Research Institute (TARI), Box 1571, Makutupora, Dodoma, Tanzania; 5 Tanzania Agricultural Research Institute (TARI), Box 509, Naliendele, Mtwara, Tanzania; 6 Tanzania Agricultural Research Institute (TARI), Box 6024, Selian, Arusha, Tanzania

**Keywords:** Seed systems, Traders, Varietal turnover, Open-pollinated varieties, Demand

## Abstract

Seed systems in sub-Saharan Africa face constant challenges such as limited access to quality seeds and low varietal turnover. Despite numerous efforts aimed at formalizing seed markets, informal seed systems still dominate with 97% of smallholder farmers sourcing seeds of sorghum, common beans and groundnuts through farm-saved seed and local grain markets. Traders are deeply embedded in these local markets and are better positioned in shaping the seed demand, particularly among smallholders who buy grains as seeds from informal outlets. However, in most cases traders remain disconnected from formal breeding and seed dissemination efforts, subsequently leaving a gap between the development of improved varieties and their adoption by farmers. This scenario perpetuates the use of old varieties that are well known to traders, limiting potential benefits from genetic gains. In this study, we examine the factors that could influence traders’ decisions to sell grains of improved varieties. Data from a structured survey of 976 traders in Tanzania is used. We find that traders with higher financial capacity, stable market access, and those having strong relationships with farmers through credit provision are more likely to sell grains of improved varieties. Similarly, we find positive associations with switching to improved varieties among traders facing business challenges, accessing information through channels other than social media, and traders selling grains as seeds at sowing. In contrast, traders engaged inthe sale of highly demanded varieties are less likely to switch to new and improved varieties. Therefore, to spur the uptake of improved varieties, strengthening trader-farmer linkages, integrating traders into structured procurement systems, and enhancing trader involvement in participatory variety selection to refine existing and preferred varieties without changing them altogether are recommended. Additionally, there is need to strengthen business modeling programs to make grain traders’ businesses bankable, expand information sharing and facilitate access to credit enabling them to adequately offer technical support to thefarmers they source grains from.

**Supplementary Information:**

The online version contains supplementary material available at 10.1007/s44279-025-00413-2.

## Introduction

Seed systems in Tanzania, particularly for open-pollinated varieties (OPV) crops such as common beans, groundnuts, and sorghum, play a crucial role in the agricultural landscape. Improved seed varieties of such crops have proven effective in increasing incomes, addressing nutrition challenges, and enhancing resilience against climate change and other stresses within production environments [1, 2]. Despite these benefits, adoption rates of improved varieties for the open pollinated crops remain low due to the dominance of informal seed systems [2, 3] Reliance on informal seed systems, such as farmer-to-farmer exchanges and local markets, is inadequate as they have limited geographical reach, hindering the dissemination of improved varieties [3].

In Sub-Saharan Africa, about 97% of OPV crop seeds come from informal sources, while the formal sector (including private companies, agro-dealer networks, and Quality Declared Seed (QDS) supplies less than 3% [4]. Seeds from these informal sources are predominantly purchased from grain markets during planting seasons and farm-saved after harvest. The reliance on informal systems limits access to quality seeds, information on improved varietal traits, and market incentives for traders, ultimately constraining varietal turnover [5, 6]. Additionally, a lack of awareness about the benefits of improved varieties, particularly among smallholder farmers, impedes their adoption [7].

Grain traders (including off-takers, aggregators, and processors) uniquely influence seed choices among farmers. However, they have been ignored in the scaling initiatives with greater investment on community seed enterprises as means to increase quality seed access [8]. Beyond supplying “local seeds”, they influence farmers’ seed choices by responding to farmer preferences and shaping demand through market trends. Traders have demonstrated that varietal replacement can be rapidly accelerated by increasing the demand for improved varieties through grain markets [9, 10]. This increases the adoption of improved climate-resilient, farmer, and market-demanded varieties. Engaging traders in formal seed dissemination is crucial for facilitating varietal turnover and improving seed system efficiency [9]. By linking the grain market with the seed market, traders can foster value chain partnerships, incentivize farmers to adopt improved varieties, and encourage investment in Early Generation Seed (EGS), Certified Seed, and QDS production to ensure quality and reliable supply. Furthermore, grain traders are strategically positioned to bridge the gap between formal breeding programs and informal seed systems by enhancing information flow on improved varieties and their benefits [10].

**Fig. 1:**
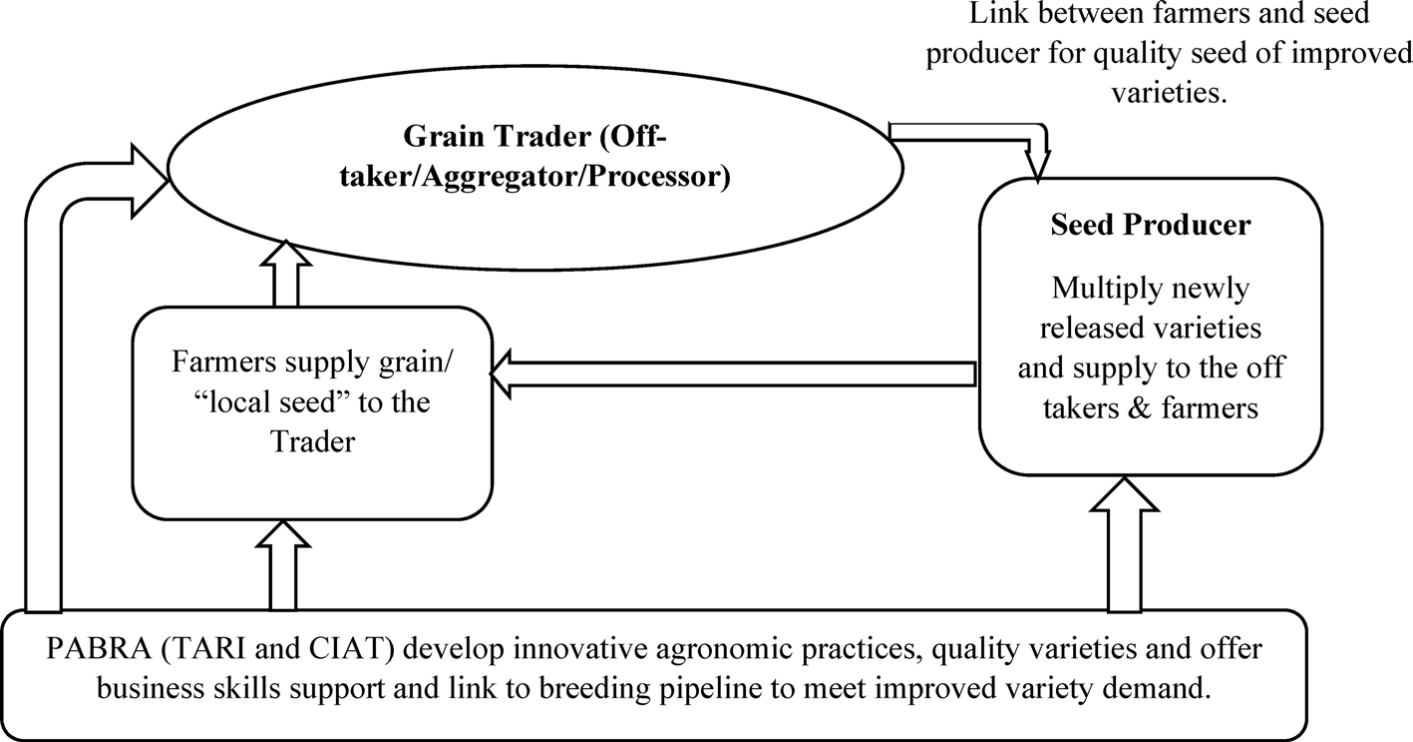
Interaction of traders with other actors to create demand and cultivate partnership. Source: Ochieng et al. [11]. TARI is the Tanzania Agricultural Research Institute, CIAT is the International Center for Tropical Agriculture, PABRA is the Pan Africa Bean Research Alliance

To better position traders to enhance seed access and accelerate varietal turnover, multi-stakeholder partnerships have emerged as a promising approach. Collaborative partnerships among key actors, including traders, farmers, and research institutions, can bridge the gap between formal breeding programs and informal seed systems [3]. As illustrated in Fig. 1, these partnerships not only enhance information dissemination but also create an enabling environment for stakeholders to engage in seed production, marketing, and distribution [9, 11]. However, the capacity of the multistakeholder platform in demand led breeding is not yet maximized as traders who are key drivers to such platforms are not adequately engaged in breeding processes. As a result of this inefficiency, there is continued dominance of proven old varieties (those that traders and farmers have sold and produced for long respectively) [2, 12].

To inform breeding and seed system priorities, this study aimed to identify the factors influencing traders’ decisions to supply grains of improved varieties of sorghum, common beans, and groundnuts in Tanzania. Specifically, it analyzed how trader characteristics, market factors, and information channels influence their willingness to switch. The study hypothesized that traders are more likely to switch to improved varieties when they perceive stronger market demand, shaped by evolving consumer preferences, and when they possess the financial and informational capacity to respond to such demand through linkages with farmers and institutions. The paper is organized as follows: the next section presents the materials and methods, detailing the theoretical and conceptual framework, data sources, and analytical approach. Section three discusses the study’s results, while section four concludes with breeding, seed systems, and market policy implications.

## Materials and methods

### Theoretical framework and analytical framework

This study is grounded in Utility Maximization Theory, which explains decision-making based on the principle of maximizing benefit [13]. In the context of this research, the traders’ decisions to switch to trading improved varieties of beans, sorghum, and groundnuts on the market, are modeled as utility-maximizing choices. Traders are assumed to switch to supplying improved varieties if the utility gained from them exceeds the utility of not-switching.

Let $\:{U}_{a}$ represent the utility derived from switching to selling grains of improved varieties and $\:{U}_{n}$ the utility from not switching. In this context, utility is not limited to profit margins but also includes perceived operational and transactional benefits and costs. For instance, the utility derived from switching may encompass access to more profitable markets, better consumer demand, and improved product traits, but also considers potential drawbacks such as additional storage, handling, or certification requirements. Conversely, (non-switching) may represent the stability of existing trade channels and lower operational risk, despite possibly lower margins. Therefore, a trader will supply the improved variety if:

1 $$\:{Y}^{*}=\:{U}_{a}-{U}_{n}>0$$

where $\:{Y}^{*}$ represents the net utility or latent benefit of switching such as access to better quality grain, higher prices of improved varieties, better processing qualities. While $\:{Y}^{*}$ is unobservable, the decision to switch $\:\left(Y\right)$ is observed and defined as:

2 $$\:Y=\left\{\begin{array}{c}1\:if\:{Y}^{*}>\:0\:\left(\text{S}\text{w}\text{i}\text{t}\text{c}\text{h}\text{i}\text{n}\text{g}\:\text{t}\text{o}\:\text{s}\text{e}\text{l}\text{l}\text{i}\text{n}\text{g}\:\text{g}\text{r}\text{a}\text{i}\text{n}\text{s}\:\text{o}\text{f}\:\text{i}\text{m}\text{p}\text{r}\text{o}\text{v}\text{e}\text{d}\:\text{v}\text{a}\text{r}\text{i}\text{e}\text{t}\text{i}\text{e}\text{s}\right)\:\:\:\:\:\:\:\:\:\\\:0\:if\:{Y}^{*}\:\le\:\:0\:(Non-switching\:to\:selling\:grains\:of\:improved\:varieties)\end{array}\right.$$

The latent utility $\:{Y}^{*}$ is determined by a combination of observed factors (e.g., perceived benefits, costs, and risks) and unobserved factors captured by an error term. The model for switching can be expressed as:

3 $$\:{Y}^{*}=X\beta\:+\:\epsilon\:$$

Where $\:X$ is a vector of explanatory variables that explain the decision to switch shown in Table 1. $\:\beta\:$ is a vector of coefficients measuring the influence of each explanatory variable. $\:\epsilon\:$ is an error term assumed to follow a logistic distribution.

Given that the decision to switch is binary, the study uses a logit regression model to estimate the probability of switching. The probability that a trader will switch to trading grains of improved varieties can be specifically tailored as:

4 $$\begin{array}{l}\:\text{log}\left(\frac{P\left(Y=1\right)}{1-P(Y=1}\right)={\beta\:}_{0}+\:{\beta\:}_{1}Sex+{\beta\:}_{1}Age+{\beta\:}_{2}Education+\:{\beta\:}_{3}{Inc+\:\beta\:}_{4}Soinfo \quad \\ +\:{\beta\:}_{5}chall+{\beta\:}_{6}PrExp+\:{\beta\:}_{7}Ttype+{\beta\:}_{8}\text{Y}\text{r}\text{B}\text{s}+{\beta\:}_{9}\text{S}\text{d}\text{c}\text{l}\text{a}\text{s}\text{s}+{\beta\:}_{10}\text{E}\text{x}\text{p}\text{t} \quad \\ +{\beta\:}_{11}\text{Q}\text{D}\text{S}/\text{T}\text{A}\text{R}/\text{T}\text{O}\text{S}\text{C}\text{I}+\:{\beta\:}_{12}\text{P}\text{t}\text{S}\text{a}\text{l}\text{e}\text{s}+\:{\beta\:}_{13}\text{L}\text{o}\text{c}\text{f}\text{a}\text{r}\:+{\beta\:}_{14}\text{M}\text{k}\text{t}\text{T}+\:\epsilon\:\:\end{array}$$

Where $\:\text{log}\left(\frac{P\left(Y=1\right)}{1-P(Y=1}\right)$ is the log-odds (degree of likelihood) of the trader switching improved varieties,$\:\:Inc\:$is the income for the trader, $\:Soinfo$ is Source of information,$\:\:chall\:$is the challenges faced by traders, $\:PrExp$ is the Prior experience of the farmer with improved varieties$\:,\:Ttype$ is the Type of the trader, $\:YrBs$ is the number of years a trader have been in business, $\:SdClass$ is the most traded seed class, $\:Expt$ is the exportation, $\:\text{Q}\text{D}\text{S}/\text{T}\text{A}\text{R}/\text{T}\text{O}\text{S}\text{C}\text{I}$ represent the linkages to $\:Quality\:Declared\:Seed/TanzaniaAgricultral\:Research\:Instit/Tanzania\:Official\:Seed\:Certification\:Institute$, $\:PtSales\:$is the point of sales or buyer category of the trader, $\:Locfar\:$is the location of a farmer, $\:MktT$ is the market type, $\:{\beta\:}_{1}-{\beta\:}_{14}$ are the parameter estimates for the explanatory variables, $\:{\beta\:}_{0}$ is the constant term and $\:\varvec{\epsilon\:}$ represents the error term.

This model was preferred over other models that are closely related such as probit regression because it provides coefficients that are easily interpretable as odds ratios, making it more practical for decision-making especially in policy and market analysis [14]. Also, while the broader seed system based on the UMT involves utility considerations across the value chain (farmers, traders, and consumers), this study focuses on utility maximization from the perspective of traders. Dimensions of trader benefit, including perceived market demand, financial and informational capacity, and linkages, are explicitly examined. The benefits to farmers and consumers are acknowledged conceptually but were not directly measured.

### Conceptual framework

**Fig. 2:**
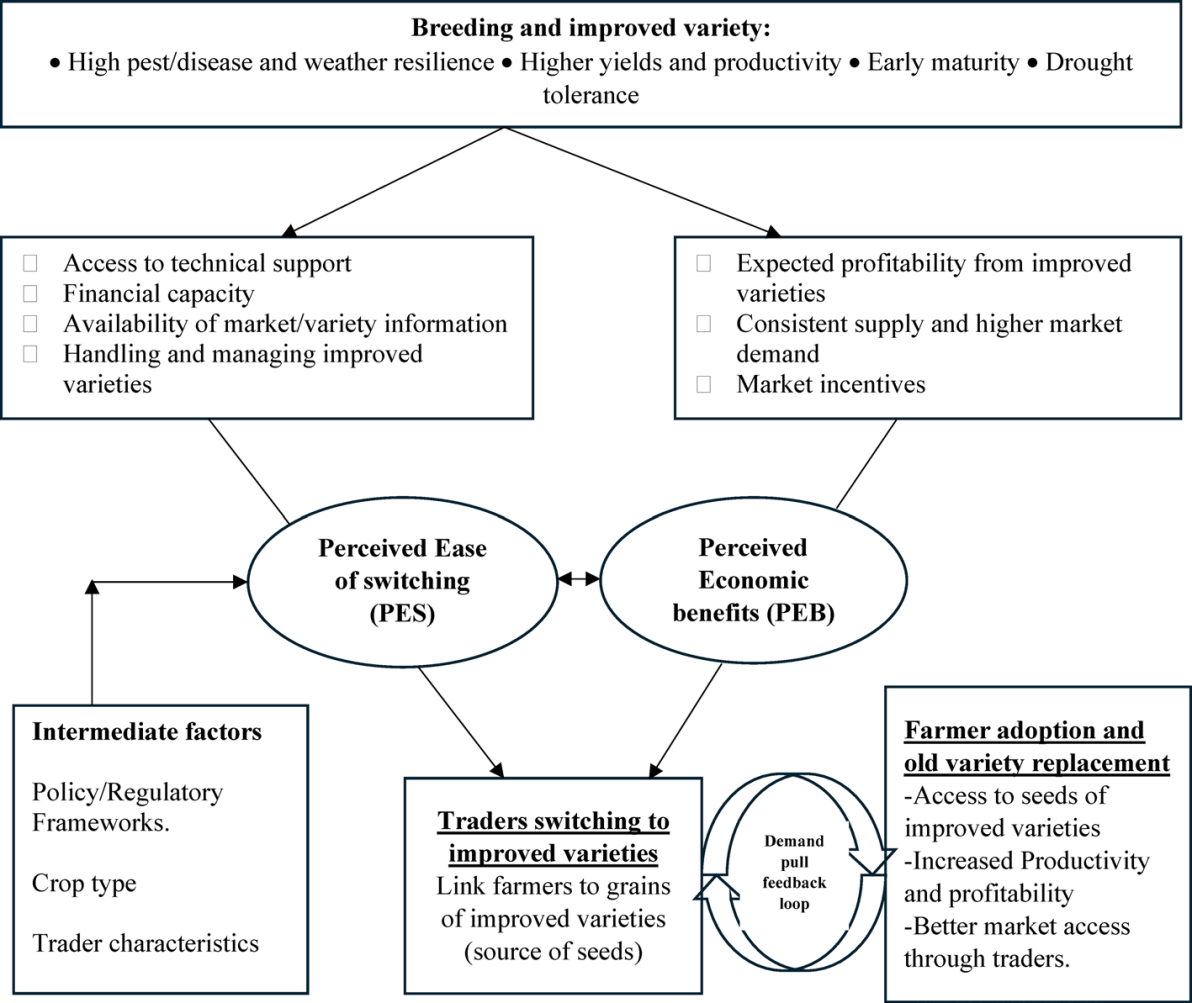
Conceptual framework of traders’ decision to switch to improved varieties. Source: Authors conceptualization

This study’s conceptual framework (Fig. 2) posits that a trader’s decision to switch to trading an improved variety is directly influenced by two primary factors that define value: Perceived Economic Benefits (PEB) and Perceived Ease of Switching (PES). PEB refers to the expected benefits traders anticipate from switching to improved varieties, such as improved market demand, consistent supply, and profitability. In contrast, the PES reflects how easily traders can incorporate improved varieties into their existing operations. They are also the costs and risks associated with switching steps. This includes factors such as access to technical support, financial capacity, availability of market information, and the ability to handle and manage improved seeds. Both PEB and PES are shaped by breeding efforts and the release of improved varieties that meet trader and consumer preferences by incorporating better traits like higher yields, greater pest and disease resistance, early maturity, and drought tolerance.

When both PEB and PES are perceived positively, traders are more likely to switch to and supply the improved varieties. Ultimately, as traders source and stock grains of improved varieties, they link farmers to improved seeds, stimulating their decision to grow improved varieties. This creates a feedback loop where farmers’ production decisions reinforce traders’ commitment to stocking improved varieties due to sustained demand and higher returns. On the other hand, farmers are assured of better access to improved seeds and market, productivity and profitability and higher returns. Policy interventions, crop type, and trader characteristics are expected to moderate the relationship between PEB, PES, and traders’ switching decisions. For example, different varieties may present varying levels of market demand, technical challenges, and profitability, influencing how traders assess the utility of adopting improved varieties.

### Trader survey

**Fig. 3:**
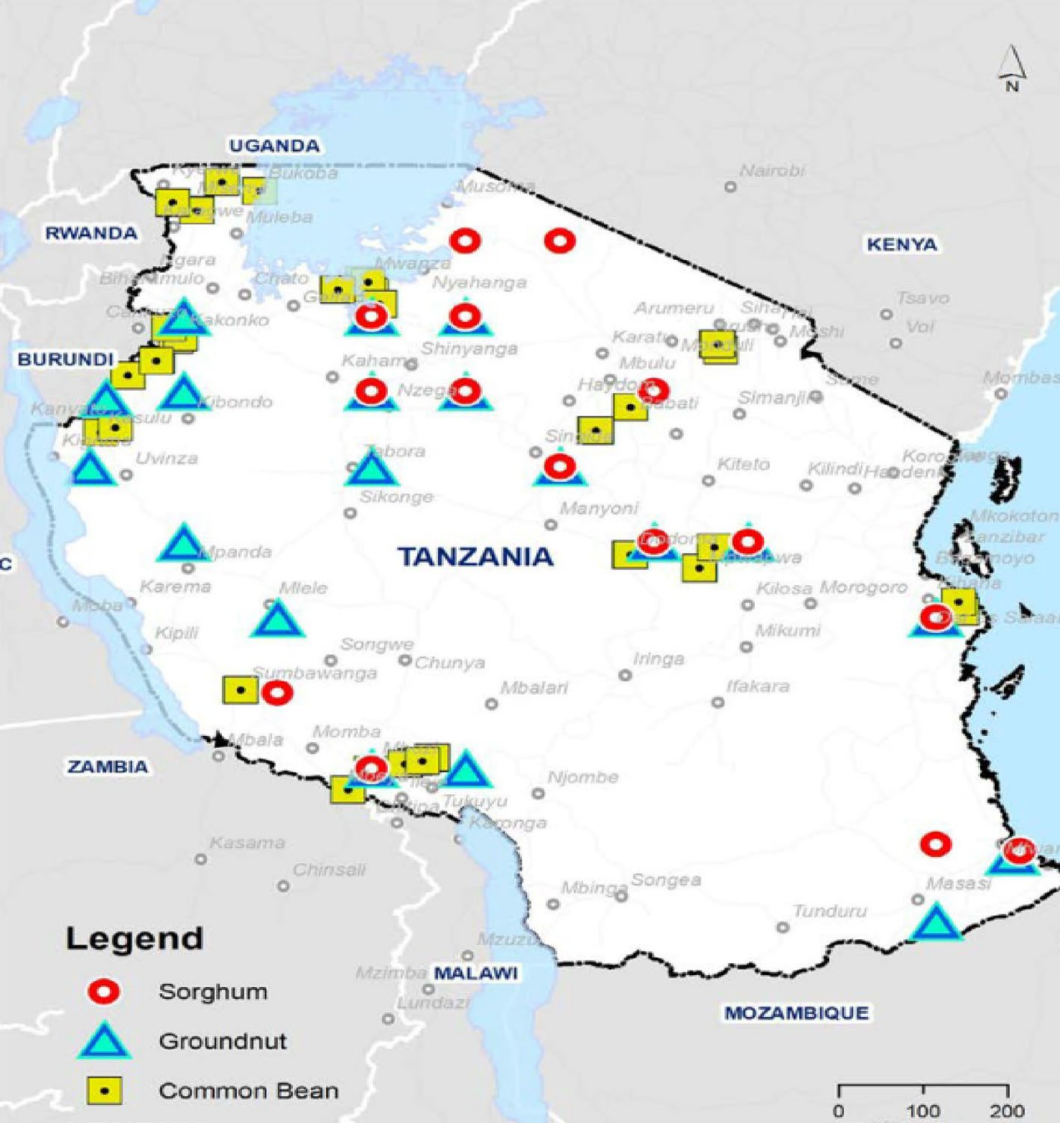
Surveyed regions

The study was conducted across all the seven agroecological zones in Tanzania namely Lake, Western, Southern, Southern highlands, Northern, Eastern, and Central zones. This covered 18 regions selected for their significance in the production and trade of beans, sorghum, and groundnuts (Fig. 3).

A multi-stage sampling approach was used, first purposively selecting key regions and markets based on the production and trade regimes for the three commodities. Secondly, 5 to 10 traders per crop from each market were randomly selected. The final sample included 976 traders, comprising 421 bean traders, 300 groundnut traders, and 255 sorghum traders. The traders were divided into large traders (off-takers) and marketplace traders.

### Data and description

Both quantitative and qualitative data were collected to assess trader incentivization to sell grains of improved varieties of the target crops. Descriptive statistics were applied to summarize trader demographics, business characteristics, and varietal preferences. Chi-square tests (χ²) assessed associations between categorical variables, such as trader types and preferred information sources. Logit regression was used to examine factors influencing traders’ willingness to switch to selling grains of improved varieties as modeled in Eq. 4. The dependent variable considered in this logit estimation was the willingness to supply improved varieties among the traders. The independent variables employed are described in Table 1.

Descriptives of major variables of interest revealed that most traders were aged 30–45 years. Male traders dominated across the three value chains (Beans = 50.59%, Sorghum = 52.94%, Groundnuts = 60.64%). Primary education was the most common form of education, particularly among sorghum and groundnuts, with minimal representation of tertiary-educated traders (Beans = 5.94%, Sorghum = 3.14%, Groundnuts = 4%).

**Table 1:** Demographic and business characteristics of grain traders, disaggregated by crop

Variable	Definition		Crop		
			Beans (*n* = 421) (%)	Sorghum (*n* = 255) (%)	Groundnut (*n* = 300) (%)
Age group	Age in years (collected recorded as categorized)	15–29	13.54	14.12	16.33
		30–45	50.59	42.75	48.00
		46–65	34.2	38.82	33.67
		66 and above	1.66	4.31	2.00
Sex	1 = male, 0 = female	Male	50.59	52.94	60.67
		Female	49.41	47.06	39.33
Education level	0 = No formal education, 1 = primary, 2 = 0-level, 3 = High school, 4 = Tertiary	No formal education	3.33	6.67	3.67
		Primary	62.95	69.80	67.00
		O-level	24.23	20.00	23.67
		High school	3.56	0.39	1.67
		Tertiary	5.94	3.14	4.00
Category/type of trader	1 = marketplace,2 = Large off-taker	Market place trader	60.57	74.12	71.00
		Large off-taker	39.43	25.88	29.00
Have sold certified seeds	1 = yes, 0 = No, 2 = Don’t know	Yes	0.24	0.39	0.33
(prior experience)		No	82.19	89.80	77.33
		Don’t know	17.58	9.80	22.33
Number of years in business	No. of years (collected recorded as categorized)	1–4 years	32.78	31.76	38.33
		4–9 years	30.88	34.51	35.00
		10–14 years	18.53	17.65	13.00
		15 years and over	17.81	16.08	13.67
Most traded seed class (top three most traded)	Common bean: 1 = Yellow, 2 = purple, 3 = Red mottled Sorghum: 1 = Red/brown, 2 = White Groundnuts: 1 = Medium tan,2 = Large red,3 = small tan	Yellow	37.65	–	
		Purple	20.11	–	
		Red mottled	18.17	–	
		Red/brown	–	50.00	
		White	–	50.00	
		Medium tan	–	–	36.99
		Large red	–	–	23.51
		Small tan	–	–	16.38
Sales outside Tanzania (exportation)	1 = yes,0 = No	Yes	12.61	15.03	12.14
		No	87.39	84.97	87.86
Linkage to QDS/TOSCI/TARI (in terms of communication)	1 = QDS producer, 2 = TOSCI, 3 = TARI	QDS	100	100	100
		TOSCI	99.76	100	100
		TARI	99.76	100	100
Market orientation (buyer category)	1 = individual, 2 = Institutions, 3 = Traders, 4 = Processors, 5 = Grains as seeds	Individuals	78.66	83.16	80.92
		Institutions	8.29	1.55	0.58
		Traders	66.05	38.60	55.49
		Processors	0.00	17.10	17.53
		Grain as seeds	36.24	37.05	42.77
Location of farmers	1 = within district, 2 = within region, 3 = other regions	Within district	44.85	62.40	44.34
		Within region	35.50	19.83	42.08
		Other regions	19.65	17.77	13.57
Support to farmers (in terms of credit offer)	1 = yes, 0 = No	Yes	19.24	16.47	15.67
		No	80.76	83.53	84.33
Income level	Monthly gross earnings (not adjusted for cost) from business sales in numbers (collected recorded as categorized)	< TzSh500000	63.90	74.12	67.33
		>TzSh500000	36.10	25.88	32.67
Source of varieties	Own production		8.47	4.92	5.01
	Direct from farmers		55.64	62.69	42.58
	Other traders		42.86	25.13	32.56
	Collectors/aggregators		33.07	36.01	43.55
	Importers		0.44	0.26	0.58
Market type	1 = urban, 2 = Rural, 3 = Border	Urban	67.70	57.65	79.00
		Rural	29.45	42.35	18.00
		Border	2.85	–	3.00

*QDS* is Quality Declared Seeds, *TARI* is the Tanzania Agricultural Research Institute, *TOSCI* is the Tanzania Official Seed Certification Institute; 1USD = Tsh 2342

Most traders (above 60%) operated as marketplace, while large off-takers comprised a smaller share of between 25% and 39% across the three crops. Business experience was concentrated in the 1–9-year range, with over 30% of traders across all crops falling within this category. Experience in certified seed sales remained rare, with over 77% of traders lacking prior experience. Regarding market type, most traders concentrated in urban centers with minimal number at the boarders. Export activity was low across all crops, with only 12–15% of traders selling outside Tanzania. The primary market orientation was toward individual consumers (78-80%), while institutional buyers remained a minor market segment (<8%). Financially, most traders operated on a small scale, with over 74% of earnings below Tsh 500,000 (USD 192). Credit provision to farmers was limited, with fewer than 20% of traders offering financial support. Seed variety demand and preferences also varied with yellow beans largely demanded (37%). Sorghum types mostly demanded were evenly split between red/brown and white types (50% each). Medium tan and large red were the dominant groundnut varieties (36%). Given the institutional critical role in seed markets, traders demonstrated near-universal awareness and interactions with QDS, TARI, and TOSCI.

## Results and discussion

### What do traders want in terms of the desired varietal characteristics

To understand the targeted points for incentivizing traders, the desired varietal characteristics were assessed, and the results are shown in Table 2. Yield emerged as the most critical and desired trait across all three crops. In addition, for beans, traders prioritized disease resistance, shorter cooking time, and improved taste. For sorghum, on top of yield, traders prioritized size and shorter maturity periods as some of the top critical traits. Regarding ground nuts, increased oil content was the most desired characteristic, followed by yield and large-sized nuts. Taste was also cited as a desired trait. These traits reflect the varied roles traders play in value chains, often balancing upstream farmer production factors (e.g., yield, disease resistance) with downstream consumer demand (e.g., taste, cooking time) and processor requirements (e.g., oil content, ease of shelling). Several attributes such as grain size, appearance, and storability also serve as marketability cues that traders use to signal quality and negotiate better prices.

While bird resistance is a major production challenge in sorghum, especially at the farm level, none of the traders mentioned it as a priority trait. This may be due to their market-oriented perspective, where traits affecting grain size, maturity, and appearance are more immediate concerns than field-level losses. These results support the hypothesis that varietal sourcing behavior is shaped by product-specific trait preferences among traders, with notable variation across crops and market segments. These differences reinforce the hypothesis that varietal sourcing behavior is highly influenced by product-specific market traits.

However, regarding the preferred traits, the study did not explicitly capture how traders weight these traits when evaluating or sourcing varieties. Nor were traits categorized by their primary value chain relevance (i.e., farmer, processor, consumer). These represent areas for further research, particularly in understanding how traders translate traits into actionable product profiles during variety sourcing.

**Table 2:** Parameters prioritized by traders for improvement in bean, sorghum, and groundnut varieties

Varieties	Beans (%)	Sorghum (%)	Groundnuts (%)
Taste	22.05	20.47	27.75
Yield	42.77	26.42	38.34
Disease resistance	30.51	16.58	24.28
Drought tolerance	–	13.47	18.50
Cooking time	23.10	–	–
Flatulence	19.49	–	–
Good size	–	23.83	–
Large	18.08	–	34.30
Small	1.50	–	0.96
Good color	–	17.10	–
Light	4.94	–	7.51
Dark	4.67	–	2.70
Processing qualities	–	18.13	23.70
Shorten maturity	–	23.32	–
Increase oil content	–	–	39.50
Reduce oil content	–	–	1.54
Easy shelling	–	–	19.27
Others^a^	19.75	10.62	7.13

^a^Others include thick broth, suitability for accompanying with other foods

### Where do traders obtain information on seed varieties (information source of different varieties)

Sources of varietal information were disaggregated by trader type to examine how market position influences access to and use of information (Table 3). Marketplace is used to refer to traders with small operations based in the marketplaces and typically procure their supply informally from walk-in farmers, brokers, or through opportunistic market interactions, while large traders operate on a large scale and are more likely to source directly from organized farmer groups, aggregators, or institutional channels, often through pre-arranged contracts or structured partnerships. For beans, both marketplace traders and large-scale traders rely mostly on other traders and farmer groups for information. However, large off-takers use social media to a greater extent in sourcing for varietal information. Notably, seed companies, agro-dealers, and customers were among the least cited sources, suggesting their limited perceived value across player types. Since both trader types rely on farmers and other traders to receive information about improved varieties, thus providing adequate information to these groups would enhance traders’ knowledge. These findings align with sperling et al., who highlight the role of informal networks in commodity value chains [4].

Like common bean traders, sorghum traders mainly use social media and farmer groups in sourcing information. However, marketplace traders also use agro-dealer shops as sources of information, while large off-takers place more emphasis on extension officers. Relative with other crops, sorghum information sourcing is also more farmer-dominated with less reliance on peer traders. This localized sourcing behavior implies less institutional or intermediary filtering of information, especially for marketplace traders. This is consistent with findings of the growing influence of social media in agricultural marketing [15].

For ground nuts, the preferred sources of variety information were fellow traders, farmer groups, agro-dealer shops, and social media. Again, seed companies and customer feedback remained less important across grain trader types. This highlights the key role of farmer groups and trader networks in sharing knowledge that emphasizes the importance of local networks in agricultural markets. The increased use of social media by large off-takers suggests its potential as a modern tool for accessing market and technical information.

Chi-square results further confirm statistically significant variation in the distribution of information sources by trader type within each crop. These findings partially reflect the broader hypothesis that traders’ decisions are influenced by access to information.

**Table 3:** Sources of variety information of beans, sorghum and groundnuts

Sources of information	Beans		Sorghum		Groundnuts	
	Marketplace trader % (*n* = 255)	Large off-takers % (*n* = 166)	Marketplace trader % (*n* = 255)	Large off-takers % (*n* = 166)	Marketplace trader % (*n* = 255)	Large off-takers % (*n* = 166)
Social media	22.4	31	42.3	57.6	5.6	11.5
Extension officers	16.7	15.2	4.2	7.6	7.5	6.9
Farmers	30.9	40	34.9	19.7	33.3	19.5
Seed companies	2.4	2.1	2.1	0	3.3	0
Agro dealer shops	4.8	6.2	7.9	1.5	10	4.6
Other traders	38.1	38	3.2	6.1	35.2	52.9
Customers	4	5	1.6	0	1.4	1.2
Others^a^	11.1	7.6	5.2	7.6	2.8	3.5
Chi-Square test	Pearson chi2(7) = 15.2485, Pr = 0.033**		Pearson chi2(7) = 13.7143 Pr = 0.057*		Pearson chi2(7) = 17.3196 Pr = 0.015**	

^a^Others include general marketplace, family and friends, agricultural shows, radio, and television. *, **, *** Significant at 10%,5% and 1% respectively; Chi-square tests compare source distribution between trader types per crop

### Technical support required by traders to promote and supply new varieties

Among the traders who cited willingness (measured against perceived net benefit from new varieties: better prices, increased demand etc.) to offer improved varieties, results show distinct technical support needs for switching and promoting grains of improved varieties (Table 4). The most frequently cited requirement was access to information about improved varieties. Reliable information is essential for informed decision-making which directly influences adoption rates [4]. This finding shows that traders often don’t have enough information about the improved varieties, yet farmers rely on them for support. Without adequate information, Ochieng et al. [16] noted that the information they give to farmers could be misleading. The need for business and management training was another priority, especially for bean traders. Chitete et al. [17] underscore the importance of business skills as an enhancer of market competitiveness and inventory management. Additionally, supplier linkage was highlighted by traders across the three crops as an important need. Viable enterprises often require the need for stable supply chains [12]. With dwindling supplies of merchandise, traders would certainly switch to more readily available products. Groundnut traders remarked on the need for extension services to address field-level challenges experienced by the farmers that supply them with grains. Others cited promotional training to improve their marketing skills. For sorghum, traders emphasized the need for promotional tools like seed leaflets to enhance farmer awareness. These findings reveal that the informational ecosystem is not neutral; instead, gaps in training and supplier linkages may constrain the ability of willing traders to adopt and promote improved varieties. This supports the hypothesis that adoption is not merely a function of preference, but also of enabling support.

**Table 4:** Technical support required by traders to sell grains of improved varieties

Nature of technical support needed by traders	Beans % (*n* = 115)	Groundnuts % (*n* = 95)	Sorghum % (*n* = 77)
Training in business and management skills	24.31	8.80	9.30
Information about improved varieties	37.78	58.2	61.3
Information on market	–	1.10	1.30
Linkages to suppliers	9.03	9.90	12.0
Extension skills	4.17	16.5	12.0
Training in sales and promotion	–	5.50	2.70
Seed promotion leaflets to give to farmers	2.78	–	1.30
Differentiating seed from grain	4.17	–	–
seed business registration and certification	2.78	–	–

### What could deter traders from switching to improved varieties?

Traders reported several challenges currently affecting their business operations which could affect their desire to use improved varieties (Table 5). Inadequate supply was the most frequently stated issue across the three crops. This concern aligns with the sourcing patterns shown above (Table 1), where most traders rely on farmers whose production is often limited by climate and input access. Such dependence may explain the reported supply inadequacy for improved varieties. Such reliance on vulnerable sources likely contributes to the supply challenges and highlights broader systemic weaknesses in seed and grain production and distribution [18]. Market instability was another major concern among beans, groundnuts, and sorghum traders with a significant number of traders citing unstable markets as a barrier to smooth trade. Additional challenges varied by crop but included poor grading, credit constraints, and inadequate market information, which limit traders’ efficiency and profitability. Groundnut traders also reported low prices, transportation costs, and inadequate storage technologies, while sorghum traders cited inadequate demand and issues related to climate variability affecting supply.

Traders’ willingness to promote and supply improved bean, sorghum and groundnut varieties is often shaped by practical business considerations such as consistent supply, grain quality, market responsiveness, and profitability [19]. Also, as shown in the disaggregated analysis by trader type (Table 1a supplementary material), both marketplace traders and large traders or off-takers report similar challenges, particularly around supply instability, low prices, and quality-related concerns, suggesting these constraints are widespread and not limited to a specific market segment. It is worth noting that, although the survey did not directly measure cost structures or profit margins, these reported constraints, particularly low product prices, high transportation costs, and inadequate storage, are indicative of pressures that could erode trader profitability.

To validate the effects of these challenges on willingness to supply new improved varieties, and considering the four components, correlation was employed as shown in Table 5. The results support that where the supply is uncertain or irregular, traders are less inclined to commit to new varieties. Similarly, concerns around mixed grains, a key indicator of grain quality, appear to discourage variety uptake, reinforcing the importance of meeting buyer expectations. On the other hand, traders who report low prices or inadequate market information show a tendency to engage with new varieties, possibly as a strategy to differentiate their products, improve their margins, or respond more effectively to market shifts. Even where demand is perceived to be low, some traders still demonstrated interest in offering new varieties, potentially in search of niches or to stimulate uptake.

**Table 5:** Major challenges faced in business that could affect the uptake of improved varieties

Type of challenge	Beans % (*n* = 421)	Groundnuts % (*n* = 300)	Sorghum % (*n* = 255)	Correlation with willingness to supply grain of improved varieties
Inadequate supply of grains	25.18	18	25.49	− 0.1601 ***
Lack of a stable market	16.15	19.33	19.22	0.0391
Inadequate demand	10.69	9.67	9.41	0.0607*
Credit constraints	7.6	8.33	3.53	0.0159
Poor grading and sorting	7.36	7.67	5.1	− 0.0370
Low Prices	4.99	6.67	5.88	0.0876***
Inadequate market information	4.51	1.67	3.92	0.0778**
High taxes/levies	3.33	4	1.18	− 0.0426
Mixed grains	1.66	2.33	1.96	− 0.0100*
Weather/climate-related challenges	1.19	0.67	2.35	− 0.0077
Delayed payments	1.19	0.33	–	0.0261
Inadequate/poor storage	1.19	2.33	3.53	0.0209
Buyers are not trustworthy	0.95	0.67	0.39	− 0.0123
Costly transportation	0.24	2.33	–	0.0225
Thefts	0.24	0.33	–	− 0.0577*
Other specify*	4.04	6.33	3.92	0.0444
No other challenges	9.5	9.33	14.12	− 0.1601***

***, **, *Significant at 1%,5%,10% respectively. *Others specified include factors around logistics & market operations, cross-border issues, trade levies and permits

.

### Factors likely to influence the trader’s willingness to switch to selling grains of improved varieties

The parameter estimates of the likelihood of switching to improved grain varieties across the three crops is presented in Table 6. Separate models were estimated for each crop to account for structural differences in markets and trader decision-making behavior. Trader type was included as a covariate to allow analysis of its influence on willingness to supply improved varieties. Results show relatively similar outcomes across the three crops with few exceptions.

For beans, the results show that the type of trader is associated with a strong likelihood of switching choices among the traders. As shown in Table 6, large off-takers are more likely to switch compared to marketplace traders. This confirms that trader profiles play a crucial role in shaping decisions around varietal supply. These differences may stem from variations in scale, market reach, or access to capital and information, which influence the types of challenges traders face in maintaining efficiency. This is in line with the findings that link financial stability and distribution networks to risk absorption among large traders [17]. The effect of financial stability is well manifested in the case of sorghum and groundnuts where earning above Tsh. 500,000 (USD 192) was associated with a higher propensity for switching to improved varieties. Adams et al. [20] support that financial security allows for better risk management and ease of investment. In contrast, bean traders showed a relatively different pattern with traders earning incomes above Tsh. 500,000 (USD 192) associated with a lower likelihood of switching to improved varieties compared to those with low incomes. This outcome can be attributed to the fact that traders with low incomes could access improved varieties and technologies as a pathway to more income, thus having a high propensity to switch.

Prior experience handling “certified seeds” was also found to be associated with a higher likelihood of switching decisions, particularly for beans and ground nuts (see Table 6). Traders without prior experience showed less likelihood of switching to improved varieties relative to those who have previously dealt with “certified seeds.” Traders who have supplied certified seeds before are likely to be more established and have market confidence coupled with prior knowledge of handling and sourcing for a market for improved varieties. Findings on the years of business showed that mid-career traders in groundnuts (4 to 14 years) display the most flexibility, while well-established traders (15 years and over) preferred proven models that have sustained their business over time. Mid-career traders often strive to balance adaptability and stability and especially try out what would work for them, thus having a greater propensity to try out improved varieties.

The type and source of information was also associated with positive odds of switching to improved varieties across the three crops. Bean traders relying more on farmer groups had a higher association with switching and offering improved varieties than those using social media. A similar pattern of positive association was observed among sorghum and groundnuts traders who sourced information from agro-dealers, farmer groups, and extension officers compared to those relying on social media. While the use of social media is mushrooming as a source of information, this outcome can be attributed to the chances of mistrust and unverifiable information shared through such platforms. Smith [21] also supports that while digital platforms such as social media are increasingly used for agricultural information, they may lack the depth and credibility required for traders to make confident decisions regarding improved varieties. Abdul-Rahaman and Gambrah [22] also adds that there is a high reliance on peer networks in the passage of agricultural information.

The age of the traders, particularly among beans and groundnut traders, was associated with the likelihood of switching. Being an older bean trader (46 to 65 years) is associated with a lower likely to switch to improved varieties compared to being younger. This finding suggests that older traders are risk averse consistent with the findings of Chikuta et al. [23]. In contrast, ground nuts findings revealed a positive association between older traders (46–65 years) and a higher likelihood of switching than young traders. Young traders are probably less likely to engage with improved varieties due to limited market experience and financial capacity. The analysis is supported by the finding that traders below 45 years earned less income compared to older traders. Further, older traders are more likely to benefit from stronger networks and greater market insights.

Market demand and seed class preferences equally played a significant role in switching decisions. Dealing with yellow beans was found to be associated with a lower likelihood to offer improved varieties than those dealing in red-mottled and purple bean classes. Yellow beans stand out as one of the most demanded bean grains within Tanzania, and so traders are largely driven to supply such a seed class. Similar trends emerged in groundnut markets, where traders supplying high-demanded seed classes (medium tan and large red) were more hesitant to adopt improved ones than those who traded small tan groundnuts. Kilima and Bolle [24] support these findings by noting that demand-driven varietal preferences influence decision-making, with most demanded varieties exhibiting stronger resistance to changes.

The geographical location of business operations also had a significant association with the likelihood of switching with bean traders in border markets more likely to adopt improved varieties than those operating in the urban areas. This outcome can be attributed to the exposure to diverse grain preferences across regions that create demand for more appealing varieties. Traders who are in rural areas largely sell to the occupants with limited preferences. This trend was different among sorghum traders, where trading in rural areas was associated with an inclination to engage with improved varieties. According to Florez et al. [25], rural regions heavily rely on informal seed systems and are great suppliers to urban regions of agricultural products. Therefore, rural traders may likely switch to improved varieties that suit agricultural zones to sustain production with prevailing environmental stressors. Further to this, the location of farmers that supplied the commodity substantially determined traders’ propensity to supply improved varieties. For beans, selling to farmers in other regions in the country was associated with a lower likelihood of switching to improved varieties than when traders sold to farmers within their district. In contrast, groundnut traders who sold to other regions and within their region showed a higher likelihood of supplying improved varieties than those who sold within their districts.

Having support mechanisms such as credit provision to farmers was positively and significantly associated with switching behavior across the three crops. Traders who provide support, especially those who extend credit, were more likely to switch to supplying improved varieties than those who did not offer credit. The provision of credit to farmers fosters trust and ensures repeat business. Consequently, with sales levels assured, they can easily introduce improved products, compared to ones with no good relations with their customers. This finding is consistent with findings by Dessalegn et al. [26] and Cacho et al. [6] who found that financial support is a key driver to adoption. This implies that the support received by traders is likely to be extended to their customers (who are largely farmers) in terms of credit.

Challenges experienced in line with business operations, especially in the post-COVID-19 era, were associated with the switching decisions among traders. Particularly, sorghum and groundnut traders who experienced various challenges were more likely to switch their grain supply than those who reported no significant challenges. Improved varieties and other technologies have the potential to increase incomes and livelihoods among those involved [1]. Traders would see this as promising improved ventures that cushion them against challenges experienced in their businesses.

Lastly, the selling point was associated with switching decisions across the three crops. Results showed that bean traders selling to institutional buyers were less inclined to offer improved varieties than those selling grains as seeds at sowing. Similarly, sorghum and groundnut traders selling to individuals, institutions, and other traders were less likely to switch than traders selling grains as seeds at sowing. With improved seeds that guarantee yield and quality harvest, traders are inclined to experience traffic at sowing time. Thus, it is likely that during this time they will have more stock of the most demanded seeds.

**Table 6:** Logit estimates for factors affecting willingness to offer (switch to) grains of improved seed varieties

Variable	Beans	Sorghum	Groundnuts
Sex of the trader (male)	0.044 ± 0.269	0.470 ± 0.332	0.737 ± 1.001
Education level (base = no formal education)			
Primary	0.405 ± 0.613	2.414 ± 1.013**	− 11.001 ± 8.152
O-level	0.014 ± 0.644	1.117 ± 1.063	− 8.644 ± 8.148
High school	0.653 ± 0.929	–	− 8.949 ± 8.453
Tertiary	− 0.088 ± 0.714	0.198 ± 1.418	–
*Age group (base = 30–45)*			
15 to 29	0.138 ± 0.365	0.636 ± 0.507	− 4.468± (1.380)***
46 to 65	− 0.617 ± 0.282**	0.264 ± 0.354	2.672 ± 0.875***
66 and above	− 0.242 ± 1.1049	1.173 ± 0.890	–
Type of trader (large off taker)	1.233 ± 0.322***	0.036 ± 0.583	1.305 ± 1.252
Income level (above Tsh 500000)	− 0.489 + 0.286*	1.707 + 0.530***	6.826 ± 1.453***
Prior experience with certified seeds (no experience)	− 1.313 + 0.365***	− 0.067 ± 0.501	− 13.887 + 2.294***
Challenges in business operations (yes)	− 0.210 ± 0.604	1.451 + 0.517***	6.446 ± 1.393***
Sources of varietal information (base = social media)			
Extension agents	0.004 ± 0.595	− 1.986 ± 0.840**	13.329 ± 3.588***
Farmer groups	1.022 + 0.456**	− 0.514 + 0.362	7.202 ± 1.248***
Seed companies	1.208 + 1.007	–	–
Agro dealers	0.842 ± 0.623	1.751 ± 0.711**	12.462 ± 2.626***
Traders	0.395 ± 0.457	− 2.675 ± 0.874***	–
Others^a^	0.151 ± 0.724	− 3.357 ± 0.783***	–
Most traded seed class (base = red mottled, small tan)			
Yellow	− 0.832 + 0.298***	–	–
Purple	− 0.438 ± 0.325	–	–
White	–	− 0.606 ± 0.395	–
Medium tan	–	–	− 10.728 ± 1.582***
Large red	–	–	− 3.642 ± 0.929***
Years in business (base = 1–4 years)			
4–9 years	0.077 ± 0.282	− 0.553 ± 0.375	3.920 ± 0.991***
10–14 years	0.264 ± 0.371	− 0.150 ± 0.483	7.592 ± 1.434***
15 years and over	− 0.113 + 0.372	− 0.467 + 0.557	− 4.863 + 1.679***
Exportation (yes)	0.185 + 0.346	0.734 + 0.530	− 0.347 + 0.925
Market type (base = urban)			
Rural	0.074 + 0.277	1.317 + 0.398***	0.629 + 0.925
Border	1.663 + 0.841**	–	–
Support to farmers (credit offer)	2.479 + 0.476***	3.014 + 0.635***	5.962 + 2.101***
Location of farmer (base = within district)			
Within region	0.059 + 0.272	− 0.355 ± 0.457	5.975 + 1.149***
Other regions in the country	− 0.563 + 0.307*	− 0.806 + 0.441	6.568 + 1.267***
Linkages with seed QDS_TARI_TOSCI (Base = QDS)			
TARI	− 0.057 + 0.259	0.173 + 0.327	–
TOSCI	− 0.196 + 0.258	0.100 + 0.325	–
Buyer category (selling point) (base = grain as seeds at sowing)			
Individual buyer	− 0.490 + 0.370	− 0.464 + 0.353	− 5.152 + 1.268***
Institutional buyer	− 2.748 + 0.899***	− 1.861 + 1.210	–
Other traders	–	− 2.183 ± 0.596***	− 4.911 ± 1.272***
Constant	0.311 ± 1.032	− 3.201 ± 1.197***	− 0.697 ± 8.419
Wald (X^2^)	87.01***	74.88***	98.91***

Standard errors in parentheses, ***, **, * significant at 1%,5%,10% respectively

^a^Others include general marketplace, family and friends, agricultural shows, radio, & television

## Lessons from other regions on accelerating varietal turnover

Experiences from outside sub-Saharan Africa offer valuable lessons on how structured seed systems, participatory breeding, and market-aligned traits can accelerate varietal turnover. In Bangladesh, the adoption of BRRI dhan71, a drought-resilient rice variety, rose to 76% within three years of release in drought-prone districts, supported by strong extension efforts and direct farmer engagement [27]. Similarly, hybrid maize adoption in India expanded rapidly in marginal areas due to public-private partnerships that linked demand-led breeding with seed supply systems [28]. These cases, even though they are different value chains, demonstrate that where enabling systems align, uptake of improved varieties can be both rapid and sustained. For Tanzania’s open-pollinated crops, such models underscore the potential of integrating traders into demand-responsive seed systems and tailoring breeding pipelines to real-world demand.

### Conclusion and policy implications

The formal seed system in Tanzania remains underdeveloped despite its high potential, especially with ongoing efforts to promote the uptake of improved varieties and related technologies. Improved varieties may face barriers limiting their adoption (at farm and market level) which affects the full realization of the goals for which they are released. Generally, newly released improved varieties face low adoption among beneficiaries (traders and farmers). Traders, who could play a key role in accelerating their uptake, are not adequately engaged, creating a gap in the system. This study analyzed the determinants that could incentivize traders to supply grains of improved varieties, ultimately promoting the adoption of these technologies among farmers. The findings largely support the hypothesis, demonstrating that traders with stronger financial capacity, prior seed handling experience, access to non-social media information sources, and established linkages with farmers and seed institutions are more inclined to supply improved varieties. While traders are positioned to influence what products reach the market, the current findings show that their product choices are still largely shaped by what farmers grow. The frequent trader–farmer exchanges observed, particularly among marketplace traders, are primarily transactional, focused on grain quality, availability, and price, with limited evidence of planning or trader influence over varietal choices by farmers. This reveals a gap in the system: the absence of coordinated mechanisms or incentives to enable traders to influence farmer production decisions. Addressing this would require closer alignment between traders, extension systems, and research institutions to ensure that information on new varieties reaches both farmers and traders in a timely and actionable manner.

Therefore, to accelerate the supply of improved grain varieties, the first approach would be to leverage the market influence of large off-takers in seed supply chains. This can be achieved by gradually strengthening their linkages with seed companies through targeted programs and promoting trader-led seed production models to enhance supply efficiency, especially in areas where current supply chains are weak or underdeveloped (as shown by the limited role of agro-dealers in the study). Another way is to integrate local traders and Quality declared seeds (QDS) producers into structured procurement systems, such as government tenders and institutional contracts, to create stable demand and encourage the adoption of improved varieties by farmers. These would complement and not replace the role played by traders by extending structured sourcing into areas they may not effectively reach. Such structured systems can also be augmented with pre-booking or pre-ordering mechanisms which subsequently ensure seed supply predictability.

Another intervention is to strengthen information dissemination by providing compelling and timely varietal information through social media and digital platforms, alongside increasing the number of extension officers who directly engage traders and agro-dealers. Specifically, Tanzania Agricultural Research Institute (TARI) and Tanzania Official Seed Certification Institute (TOSCI) need to have an intense extension reach to enhance awareness to improve switching or adoption rates. Once there is information reach, other action plans including streamlining seed production to align with seasons(demand) will be a sure way to ensure steady supply which in turn motivates traders. For example, availing more grains during planting (that are sold as seeds) especially at sowing and reducing market uncertainty, would make traders more inclined to transition to the abundant improved varieties.

Access to business development services is equally crucial to incentivizing traders. To meet this goal, lead organizations need to establish strong business acceleration programs that equip traders with essential business management skills, financial literacy, and tailored marketing plans. These initiatives should also focus on making traders’ businesses bankable by improving their ability to access credit and investment opportunities. Working towards ensuring financial stability among traders would help in building their relations with farmers, especially when they have the ease of extending credit.

Further, there is a need to strengthen participatory variety selection whereby improved varieties released directly match the market demand (especially traders and farmers preferences who play a central role in shaping varietal adoption). To achieve this, maintaining well-preferred seed classes while improving them with desirable traits should be prioritized. This implies that breeding efforts should ensure that the core identity of major seed classes is maintained while enhancing their performance rather than completely replacing them. This approach will ensure continuity in consumer demand while incorporating desirable agronomic and productivity traits. For sorghum, low adoption may also stem from improved varieties not meeting farmers’ multifunctional needs. As an indigenous crop, sorghum serves roles beyond food, such as brewing, feed, and construction. When improved types lack these qualities, farmers are less likely to adopt them. This underscores the need for participatory variety selection that reflects both agronomic and cultural-functional traits.

Finally, addressing the supply bottlenecks identified in this study could benefit from improved distribution systems across regions. Strengthening infrastructure and coordination in seed movement, especially where traders rely on farmer-based sourcing, would help expand access to improved varieties in areas with limited local production.

### Limitation and areas for further research

This study captures the key sources from which traders obtain varietal information, as well as the attributes they prioritize, which also allowed us to infer the kinds of information they value when sourcing for grains of improved varieties. However, the survey did not explore how traders use this information when interacting with clients, nor did it assess information-product linkages or networked relationships such as who supplies what, to whom, and with what accompanying advisory content. Future research could investigate how information is transferred across actors in the value chain, the extent to which traders influence farmer or consumer decisions, and how product flows and advisory flows are bundled or separated in practice. 

## Supplementary Information

Below is the link to the electronic supplementary material.

Supplementary Material 1

## Data Availability

Data available on request.
