# Supplementary material for: Engaging traders in strengthening seed systems in Tanzania: key drivers for selling grains of improved varieties of sorghum, common beans and groundnuts
Source: Discov Agric. 2025 Nov 10;3(1):241. doi: 10.1007/s44279-025-00413-2 (PMC12602625; doi:10.1007/s44279-025-00413-2)
Supplement: Supplementary file 1 — Supplementary Material 1 [file 44279_2025_413_MOESM1_ESM.docx]

**Annex 1: Supplementary material**

Main post Covid challenges faced in business that could affect the uptake of improved varieties, disaggregated by type of the trader

| Main challenges reported | Total | | Type of trader (%) | |
| --- | --- | --- | --- | --- |
|  |  |  | Market place traders | Large grain traders (offtaker) |
|  | Response (n) | % |  |  |
| No challenge | 104 | 10.66 | 8.83 | 14.42 |
| Inadequate supply of grains | 225 | 23.05 | 20.40 | 28.53 |
| Lack of a stable market | 175 | 17.93 | 18.87 | 15.99 |
| Inadequate demand | 98 | 10.04 | 11.26 | 7.52 |
| Poor grading and sorting | 67 | 6.86 | 7.46 | 5.64 |
| Credit constraints | 66 | 6.76 | 71.5 | 5.96 |
| Low prices | 56 | 5.74 | 5.18 | 6.90 |
| Inadequate market information | 34 | 3.48 | 4.11 | 2.19 |
| High taxes/levies | 29 | 2.97 | 3.20 | 2.51 |
| Inadequate/poor storage facilities | 21 | 2.15 | 2.13 | 2.19 |
| Mixed grains of different varieties | 19 | 1.95 | 2.44 | 0.94 |
| Weather/climate related constraints | 13 | 1.33 | 1.37 | 1.25 |
| High cost of transportation | 8 | 0.82 | 0.61 | 1.25 |
| Buyers are not trustworthy | 7 | 0.72 | 0.61 | 0.94 |
| Delayed payments | 6 | 0.61 | 0.61 | 0.63 |
| Theft | 2 | 0.20 | 0.30 | 0.00 |
| Others specified* | 46 | 4.71 | 5.48 | 3.13 |
| **Number of observations (N)** | **976** |  | **657** | **319** |

*Others specified include factors around Logistics & market operations, Cross-border issues, trade levies & permits.
